# The GUIDE-HF protocol: a randomized controlled trial of bioelectrical impedance analysis-guided diuretic therapy on prognosis in patients with acutely decompensated chronic heart failure

**DOI:** 10.3389/fcvm.2026.1798341

**Published:** 2026-05-08

**Authors:** Qilong Guo, Xinru Liu, Yajie Xu, Zukela Tuerhong, Shuai Shang, Meng Wei, Meidina Yeerken, Xiaoting Zhang, Xiaotong Liu, XingLi Gu, Xin Du, Yanmei Lu, Baopeng Tang

**Affiliations:** 1 Department of Cardiac Pacing and Electrophysiology, The First Affiliated Hospital of Xinjiang Medical University, Urumqi, China; 2 Department of Cardiology, The Affiliated Hospital of Qingdao University, Qingdao, China; 3 Department of Cardiology, Capital Medical University Affiliated Anzhen Hospital, Beijing, China; 4 Xinjiang Key Laboratory of Cardiac Electrophysiology and Cardiac Remodeling, The First Affiliated Hospital of Xinjiang Medical University, Urumqi, China

**Keywords:** acute decompensation of chronic heart failure, bioelectrical impedance analysis, diuretic therapy, fluid overload, heart failure, patient readmission, randomized controlled trial

## Abstract

**Background:**

Patients with acute decompensation of chronic heart failure often have residual fluid retention at hospital discharge, which is closely associated with high rates of rehospitalization and mortality during the post-discharge vulnerable phase. This “revolving door” phenomenon imposes a substantial economic burden on healthcare systems and contributes significantly to inpatient bed occupancy. Current clinical assessment methods are highly subjective and lack precise quantitative indicators. This study protocol proposes a randomized controlled trial aiming to use accurately quantifiable fluid overload measured by Bioelectrical Impedance Analysis-a non-invasive, cost-effective, and portable technology-to guide diuretic therapy and optimize volume management.

**Methods:**

This study is a single-center, open-label, randomized controlled trial with blinded endpoint assessment, and aims to enroll 538 participants at the First Affiliated Hospital of Xinjiang Medical University. Patients will be randomly assigned in a 1:1 ratio to either the intervention group (diuretic therapy guided by fluid overload measured using the Bioelectrical Impedance Analysis) or the control group (standard empirical treatment). The study aims to investigate the association between a volume management strategy guided by precise quantitative assessment of fluid overload and patient outcomes. The primary endpoint is the composite of heart failure rehospitalization or all-cause death within 3 months after discharge.

**Potential impact:**

The expected results of this study will demonstrate the clinical benefits of diuretic therapy guided by a precise volume management strategy, thereby improving treatment practices for hospitalized patients with heart failure and promoting the standardized application of this approach in heart failure management. At a broader level, this study provides a paradigm for exploring precision medicine in heart failure by integrating noninvasive monitoring technologies with conventional pharmacological therapy to improve key hard clinical endpoints. Ultimately, the study supports a shift in heart failure management toward a more individualized and goal-oriented approach, offering new insights and tools to reduce the burden of heart failure and improve long-term patient outcomes. The recruitment has started and is ongoing, and this protocol outlines the study's design, methodology, and implementation framework.

**Clinical Trial Registration:**

https://www.chictr.org.cn/, Chinese Clinical Trial Registry, ChiCTR2500111518.

## Introduction

Acute heart failure (AHF) is a clinical syndrome with rapid onset or sudden worsening of heart failure symptoms and signs, triggered by diverse etiologies. It is characterized by elevated plasma natriuretic peptides and often follows a life-threatening course that requires prompt medical intervention, frequently necessitating emergency hospitalization. Acute decompensation of chronic heart failure represents the predominant AHF phenotype, accounting for approximately 70%–80% of all AHF admissions, and is associated with poor prognosis. Registry data indicate that in-hospital mortality for AHF is approximately 12%, while the incidence of death or heart failure rehospitalization reaches 30%–45% within 90 days and 67% within one year (1). This high rate of readmissions places a substantial burden on global healthcare resources and leads to critical shortages in hospital bed capacity. Consequently, reducing potentially preventable readmissions has become a key priority for public health policy and hospital quality improvement programs worldwide.

This excess risk has been attributed to inadequate diuresis during index admission; over 50%–70% of patients are discharged with residual fluid overload (2–4). Conversely, intensified inpatient diuretic regimens attenuate congestion at discharge and improve post-discharge outcomes (3). Nevertheless, diuretic use and dose titration in AHF remain a double-edged sword. Early, aggressive diuresis rapidly relieves symptoms, shortens length of stay, and may reduce mortality (5). Conversely, the absence of precise guidance predisposes to over-diuresis, precipitating electrolyte disturbances, hypotension, and worsening renal function with resultant clinical deterioration (6, 7).

Determining the optimal approach to guide diuretic therapy, remove excess fluid, and improve clinical outcomes remains an unresolved challenge. Currently, traditional assessment methods, such as weight measurement, skinfold thickness, and calculation of Body Mass Index (BMI), have significant limitations in precisely determining the extent of fluid retention within the body. Established body-composition tools such as underwater weighing or dual-energy x-ray absorptiometry, although accurate, are invasive, require specialized equipment and trained personnel, and carry substantial cost, all of which preclude routine clinical use. Consequently, the pursuit of precise, quantitative, and non-invasive strategies for diuretic management continues to command intense international investigative interest.

Bioelectrical Impedance Analysis (BIA) can directly quantify fluid retention, providing a noninvasive method for precise assessment of fluid overload (8, 9). Evidence suggests that precision volume management strategies have already been integrated into the care of patients with renal dysfunction or those receiving dialysis; however, their application in the field of heart failure remains exploratory and is limited by a lack of evidence from large-scale randomized controlled trials (10, 11). Therefore, we designed a randomized controlled trial to address this gap and to evaluate the impact of diuretic therapy guided by precise fluid overload assessment on clinical outcomes in Chinese patients with acute decompensation of chronic heart failure.

## Methods and analysis

### Study objectives

The primary objective of this single-center, randomized controlled trial is to evaluate the impact of a precision fluid management strategy, guided by BIA, on clinical outcomes in patients hospitalized for acute decompensated heart failure, compared with standard empirical diuretic therapy.

The primary endpoint is the composite of heart failure rehospitalization or all-cause mortality within 3 months after discharge. The key secondary endpoints include the evaluation of individual components of the primary outcome, cardiovascular mortality, changes in quality of life as assessed by the Kansas City Cardiomyopathy Questionnaire (KCCQ) score, symptom relief measured by Visual Analogue Scale (VAS) score, and in-hospital diuretic utilization. The safety profile of the intervention, with specific regard to blood pressure, renal function, and electrolyte stability, will also be assessed.

### Study design

This investigator-initiated, single-center, two-arm, randomized controlled trial will be conducted at a tertiary hospital in Xinjiang, China to compare the incidence of composite endpoint events (heart failure rehospitalization and all-cause mortality) between the two groups over a 3-month follow-up period, thereby providing practical evidence to support the clinical application of this technology in this patient population.

**Figure 1:**
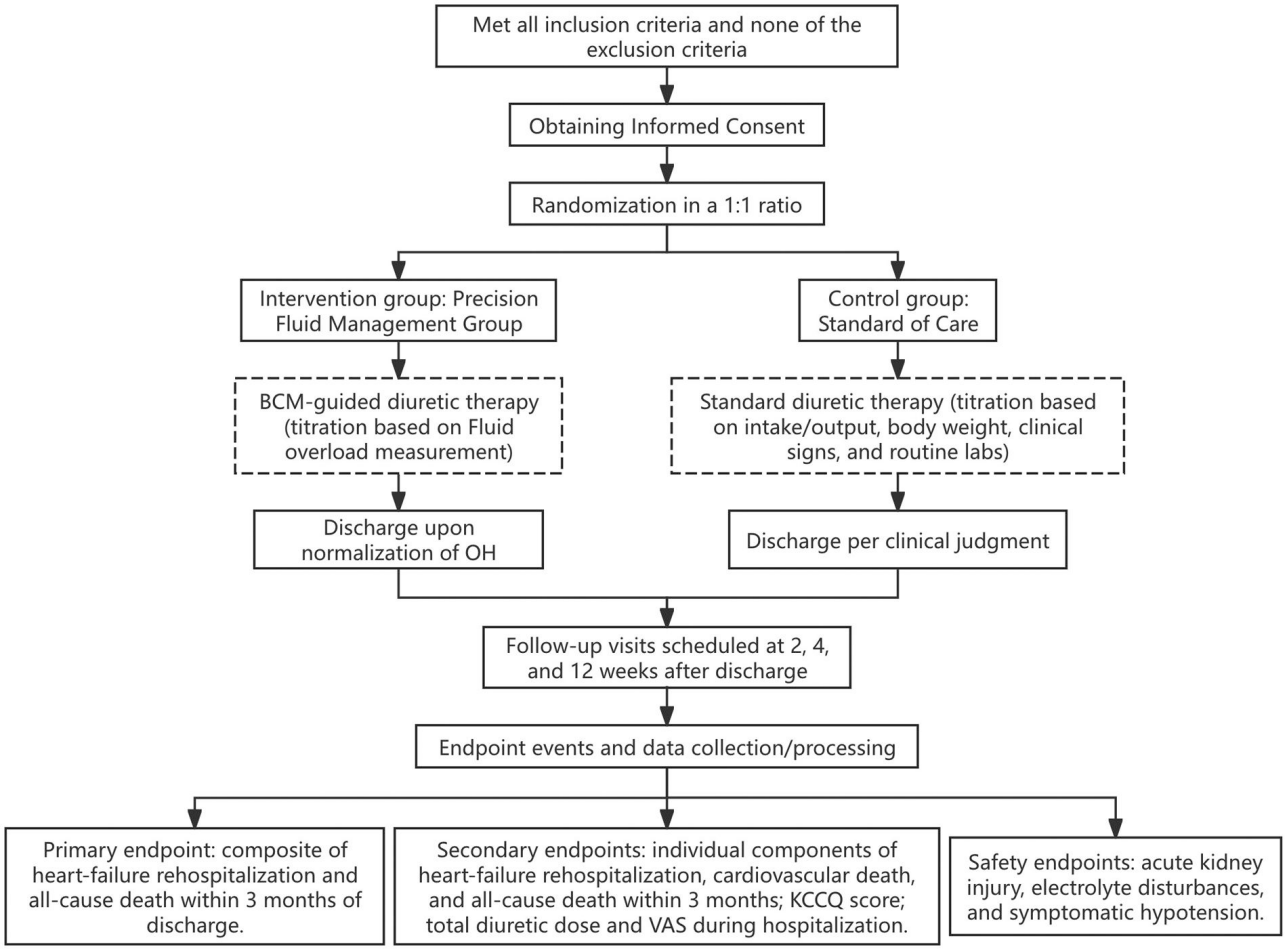
Study flow diagram of the trial. BCM, Body Composition Monitor; OH, overhydration.

A total of 538 adult patients clinically diagnosed with acute decompensation of chronic heart failure and who meet the inclusion and exclusion criteria. The intervention will be conducted during the patients’ hospitalization, and clinical assessments will be performed at 2 weeks, 4 weeks, and 12 weeks after discharge. Figure 1 shows the patient flow through the study. This study is registered at Chinese Clinical Trial Registry (ChiCTR2500111518).

### Study population and recruitment

Eligible patients who meet all inclusion criteria and no exclusion criteria will be enrolled from the Department of Cardiology, The First Affiliated Hospital of Xinjiang Medical University. Potential participants will be approached, informed, and invited by the clinical team; during this process, a physician coordinator will explain the protocol, provide the informed-consent form, and confirm eligibility on the basis of clinical status. The specific inclusion and exclusion criteria are detailed in Table 1.

**Table 1:** Inclusion and exclusion criteria.

Criteria Type	Specific Content
*Inclusion criteria*	1. Aged 18 to 75 years;
	2. Hospitalization for acute decompensated heart failure or worsening heart failure symptoms;
	3. Established prior diagnosis of chronic heart failure;
	4. Clinical evidence of fluid overload (e.g., pulmonary rales, peripheral edema, jugular venous distension, ascites, pulmonary congestion, or radiographic pulmonary edema) requiring intravenous diuretic therapy as determined by the treating physician;
	5. Provision of written informed consent.
*Exclusion criteria*	1. Have other serious diseases that may endanger survival within 3 months, and have rehospitalization plans (including revascularization, device implantation, etc.) within 3 months after discharge;
	2. The history of two or more electrolyte disturbances in the past year;
	3. Severe renal insufficiency (serum creatinine >442 μmol/L or eGFR <30 mL/(min·1.73m^2^), nephrotic syndrome or receiving long-term regular dialysis;
	4. Severe hypothyroidism and decompensated cirrhosis;
	5. Lower extremity cellulitis, severe varicose veins, lymphedema, or deep vein thrombosis;
	6. Pregnant or bedridden patients unable to take weight measurement;
	7. Patients presenting with fever or diarrhea upon hospital admission.

### Sample size

The sample size was calculated to test the superiority of a BIA-guided precision fluid management strategy over standard empirical diuretic therapy. Based on previous data, the 3-month incidence of the composite outcome of heart failure rehospitalization or all-cause mortality in the control group was assumed to be 24.9% (1). We hypothesized a 20% relative risk reduction with the intervention, corresponding to an expected event rate of 19.9% in the intervention group.

With a significance level of 0.05, a power of 80%, and a 1:1 randomization ratio, a total of 242 participants per group were required. Allowing for a 10% attrition rate, the final target sample size was increased to 269 participants per group, yielding a total sample size of 538. Sample size calculations will be performed using PASS software.

### Randomization and allocation concealment

Participants will be randomly allocated in a 1:1 ratio using a computer-generated randomization sequence with variable block sizes (e.g., 4, 6, or 8). The randomization list will be prepared by an independent statistician and concealed from investigators involved in enrollment and clinical management. Allocation concealment will be ensured via a centralized electronic randomization module within the electronic data capture (EDC) system. Randomization will be performed only after eligibility confirmation and informed consent; prior to randomization, investigators responsible for screening and enrollment will have no access to the allocation sequence or the upcoming assignment.

Given the single-center design and relatively large sample size, stratified randomization was not implemented. Baseline characteristics, including diuretic exposure, will be assessed for balance between groups. If clinically meaningful imbalance is observed, adjusted analyses will be performed.

### Blinding

This is an open-label trial with blinded endpoint adjudication. An independent Clinical Events Committee (CEC), whose members are blinded to treatment allocation, patient identity, and all fluid load data, will adjudicate all potential primary and secondary endpoint events according to pre-specified, standardized definitions.

### Interventions

#### Run-in phase

1. Instrumentation: Ensure that the Body Composition Monitor (BCM, Fresenius, Germany) used for the measurement and assessment of fluid overload is properly calibrated and maintained in optimal working condition.
2. Staff training: All healthcare personnel participating in the study will receive standardized training to ensure a thorough understanding of the study objectives, methodology, and procedures, as well as the correct use of the measurement devices and the proper interpretation of their results.
3. Patient screening and enrolment: All participants must meet the predefined inclusion and exclusion criteria, and written informed consent must be obtained prior to enrollment. Participants will be informed of the measurement procedures and any lifestyle modifications or treatment regimens required during the intervention period. Randomization will be completed within 24 h of hospital admission.

#### Intervention strategy

During hospitalization, BCM measurements will be conducted at a fixed time daily to quantify overhydration (OH) and guide daily diuretic adjustments.

Patients with OH > +1.1 L will be considered overhydrated and receive protocol-guided intensification of diuretic therapy, including an increase in the intravenous dose of loop diuretics by 25%–50% compared to the previous day. Both intermittent intravenous bolus and continuous intravenous infusion of loop diuretics are permitted during the trial, at the discretion of the treating physician. The mode of administration will be prospectively recorded and considered in exploratory subgroup and sensitivity analyses. If diuretic response remains inadequate, further escalation or the addition of predefined synergistic diuretics will be permitted. Patients with OH within the normal range (−1.1 L to +1.1 L) will receive maintenance or de-escalated diuretic therapy, with encouragement to transition to oral maintenance dosing when clinically stable (12). For patients with OH < −1.1 L, diuretic doses will be reduced by ≥50% or temporarily withheld for 24 h, and volume status will be reassessed to avoid hypovolemia.

Insufficient diuretic response is defined as failure to achieve at least one of the following within 24–48 h after dose adjustment: 1) net negative fluid balance of at least 500 mL per day, 2) body weight reduction of at least 0.5 kg, or 3) improvement in congestion-related symptoms. Clinical stability defined as relief of congestion symptoms, stable hemodynamics, and no requirement for intravenous diuretics for at least 24 h.

In cases of insufficient response, further escalation of loop diuretic dose or the addition of a synergistic diuretic agent (e.g., thiazide-type diuretics or mineralocorticoid receptor antagonists) will be permitted within guideline-recommended dose ranges, at the discretion of the treating physician.

BCM measurements will be performed using the Body Composition Monitor (Fresenius Medical Care, Germany) with standard wrist-to-ankle electrode placement according to the manufacturer's instructions. Measurements will be conducted once daily in the morning before intravenous diuretic administration. Participants will rest in a supine position for at least 5–10 min prior to measurement and will be instructed to refrain from excessive fluid intake for at least 2 h before the assessment.Overhydration (OH) values will be automatically calculated using the device's validated algorithm. Detailed measurement procedures are provided in Supplementary File S1. If a BCM measurement is not feasible on a given day, the reason must be documented, and volume management for that day will revert to protocol-defined clinical assessment criteria.

Safety triggers for de-escalation or interruption of diuretic therapy will include hypotension, worsening renal function, or clinically significant electrolyte abnormalities. In such cases, treating physicians will be allowed to deviate from the algorithm to ensure patient safety. All recommended adjustments and actual treatments will be prospectively documented, and protocol deviations will be recorded for subsequent per-protocol and sensitivity analyses.

#### Control strategy

BCM will not be used; physicians will estimate dry-weight from intake/output records, daily weight, clinical signs, and routine laboratory data to guide diuretic titration, and discharge timing will be determined at the clinician's discretion.

To minimize contamination between study groups, BCM measurements will be performed exclusively in the intervention group by independent research personnel who are not involved in routine clinical care. Participants in the control group do not undergo BCM measurement at any time during hospitalization. BCM results will not be entered into the electronic medical record and will not accessible to treating physicians caring for control-group participants.

#### Concomitant therapy

Management will adhere to international heart-failure guidelines. All participants will receive standardized education on sodium and fluid restriction, daily weight monitoring, and physical activity. Patients with HFrEF will be started as early as possible on quadruple goal-directed therapy with Angiotensin-Converting Enzyme Inhibitor/Angiotensin II Receptor Antagonist/Angiotensin Receptor-Neprilysin Inhibitor (ACEI/ARB/ARNI), Beta blockers, Mineralocorticoid Receptor Antagonist (MRA), and Sodium-Glucose Co-Transporter 2 Inhibitors (SGLT2i); vericiguat will be added when indicated unless contraindicated or not tolerated. Drugs will be initiated at evidence-based doses, uptitrated in rapid succession to target or maximum tolerated levels, prioritizing simultaneous introduction of agents with complementary mechanisms.

### Study outcomes and measurement

The primary outcome will be assessed at three predefined time points: 2 weeks, 4 weeks, and 12 weeks after discharge. Secondary clinical endpoints will be evaluated at the same time points as the primary outcome. Patient-reported quality of life measures will be assessed during hospitalization, at discharge, and at 12 weeks post-discharge. Additionally, assessments of symptom relief and safety monitoring will be conducted during the hospital stay (as detailed in Table 2).

**Table 2:** Summarizes the timing of enrollment, interventions, and outcome assessments.

Visit ID	V1	V2	V3	V4	V5	V6
Case Report Form (CRF)	A	B		C	D	E
	Screening	Baseline/Randomization	Discharge	Follow-up 1	Follow-up 2	End-of-Study Visit
Time from Randomization	-1-0 week	-1-0 week	0 week	2 week	4 week	12 week
Assessment Window	–	–		±3 days	±3 days	±3 days
Informed Consent	X					
Demographic Data		X				
Personal History		X				
Physical Examination		X				
Comorbidities		X				
Laboratory Tests		X				X
Concomitant Medications		X		X	X	X
Event Adjudication				X	X	X
Heart Failure Symptom Assessment (KCCQ/VAS)		X	X			X (KCCQ)
Bioelectrical Impedance Measurement		X (Daily during hospitalization, intervention group)				

The primary outcome of this study is the composite endpoint of heart failure rehospitalization and all-cause death within 3 months after discharge; the secondary outcomes include rehospitalization for heart failure within 3 months after discharge, cardiovascular death within 3 months after discharge, all-cause death within 3 months after discharge, the change of Kansas City Cardiomyopathy Questionnaire (KCCQ) quality of life score from baseline to discharge and 3 months after discharge, the dose of diuretics used during hospitalization and dyspnea and changes in overall condition at 72 h after admission (VAS scale); the safety endpoints include the incidence of acute kidney injury and hypotension during hospitalization, the incidence of electrolyte imbalance during hospitalization and follow-up period.

The time origin for the primary endpoint is defined as the date of hospital discharge. Participants will be followed for 90 days after discharge.

#### Endpoint definitions

Heart-failure rehospitalization is defined as an unplanned hospital admission lasting ≥24 h with a primary diagnosis of heart failure, accompanied by worsening signs/symptoms of congestion and requiring intensification of HF therapy (e.g., intravenous loop diuretics and/or vasoactive therapy). Planned admissions for elective procedures without clinical deterioration are not counted as endpoint events. All-cause death is defined as death from any cause occurring within 90 days after discharge.

Hypotension is defined as a systolic blood pressure <85 mmHg during hospitalization requiring reduction in diuretic therapy or initiation of vasopressor support.

Acute kidney injury (AKI) is defined as the occurrence of any one of the following: 1) Serum creatinine increases by ≥0.3 mg/dl (≥26.5 μmol/L) within 48 h; 2) Creatinine rises to >1.5 times baseline, indicating known or presumed renal dysfunction occurring within 7 days; 3) Urine output <0.5 ml/(kg·h) for at least 6 consecutive hours.

Electrolyte disturbances: 1) Hyperkalemia is defined as any serum potassium level >5.5 mmol/L observed during hospitalization or follow-up. 2) Hypokalemia is defined as any serum potassium level <3.5 mmol/L observed during hospitalization or follow-up. 3) Hyponatremia is defined as any serum sodium level <130 mmol/L observed during hospitalization or follow-up. Hypernatremia is defined as any serum sodium level >150 mmol/L observed during hospitalization or follow-up.

#### Event ascertainment and adjudication

Potential endpoint events will be identified through 1) in-hospital electronic medical records and discharge databases, and 2) standardized follow-up contacts at 2, 4, and 12 weeks after discharge. For hospitalizations outside the study center, participants or family members will be asked to provide discharge summaries and key supporting documents (admission/discharge notes, medication records, imaging/laboratory reports). All suspected events will be adjudicated by an independent Clinical Events Committee blinded to treatment allocation and BCM data, using prespecified definitions. Disagreements will be resolved by consensus or a third adjudicator.

After investigators submit the relevant case-report documentation, independent, blinded medical adjudicators contracted for the study will determine 3-month cardiovascular mortality, all-cause mortality, number of heart-failure hospitalizations, time to first heart-failure admission, and any associated questionnaires.

The Kansas City Cardiomyopathy Questionnaire (KCCQ) will be administered at baseline by an independent, blinded assessor on site; at the 3-month follow-up, the same instrument will be completed via telephone by an independent, blinded assessor.

### Data collection and management

Data will be entered and stored using a secure EDC system with built-in real-time validation rules. Logical checks and predefined ranges for key variables will be applied to minimize data entry errors. The EDC system will automatically generate visit schedules and allowable time windows for each enrolled participant, facilitating continuous and standardized data management.

#### Data entry

Following each subject visit, the investigator or authorized research staff will log into the EDC system and enter data based on medical records or the paper-based Case Report Form (CRF) completed during the visit, including laboratory test reports and all relevant supporting documents (e.g., admission records), to complete the electronic Case Report Form (eCRF) entry.

#### Central data-quality review

Data managers will perform routine quality control procedures, including logic checks, consistency validation, and targeted reconciliation of key variables against the CRF, to ensure data completeness and accuracy. Additionally, real-time monitoring of data collection and entry timelines will be conducted to maintain a high-fidelity study database.

#### Feedback and monitoring for clinical research center

Data verification reports will be periodically generated and provided to the clinical research center, summarizing data entry timeliness and the occurrence of missing or inconsistent data. The data collection process will undergo regular independent review, with particular attention to data completeness and quality. Any identified issues related to data collection practices will be communicated to the center for appropriate follow-up action in accordance with the study's quality management procedures.

#### Data confidentiality

All personnel (site investigators, project-management staff, and third-party vendors) are bound by strict confidentiality agreements. Paper CRFs and source medical records remain under lock at the participating sites; copies of SAE and clinical-event source documents retained by the coordinating office are stored in locked cabinets within secure, access-controlled rooms. If upload via the study web portal is required, files reside in a non-public directory reachable only through the internal project site. All database access is username/password-protected, passwords are encrypted at rest.

#### Data sharing

Deidentified participant data will be available upon reasonable request from the corresponding author, beginning 6 months after publication and for 3 years thereafter. Requests must specify the scientific rationale, variables sought, intended analyses, and analytical plan, and will be reviewed by the steering committee prior to approval and secure transfer under a data-use agreement.

### Statistical analysis

Analyses will be performed with SPSS 26.0 (SPSS Inc., Chicago, IL) and R 4.2.1 (R Foundation, Vienna). All analyses will follow the intention-to-treat principle, with participants analyzed according to the randomized assignment. Baseline characteristics will be summarized using mean (SD) or median (IQR) for continuous variables and counts (%) for categorical variables.

The primary endpoint (time to first occurrence of heart-failure rehospitalization or all-cause death within 3 months) will be analyzed using Kaplan–Meier methods and compared between groups using the log-rank test. Treatment effect will be estimated using Cox proportional-hazards models, reporting hazard ratios (HRs) with 95% confidence intervals. The proportional-hazards assumption will be assessed using Schoenfeld residuals. A prespecified adjusted Cox model will include clinically relevant baseline covariates (e.g., age, sex, BMI, LVEF, NYHA class, baseline NT-proBNP). Baseline diuretic dose (expressed as furosemide-equivalent dose) will be compared between groups and included as a covariate in adjusted analyses if clinically relevant imbalance is observed.

For time to first heart-failure rehospitalization, death will be treated as a competing event; Fine–Gray subdistribution hazard models will be used as a supportive analysis. Cardiovascular and all-cause mortality will be analyzed using KM/log-rank and Cox models as appropriate.

The total number of heart-failure rehospitalizations within 3 months will be analyzed using a negative binomial regression model (offset by follow-up time), reporting incidence rate ratios (IRRs) with 95% CIs. As a sensitivity analysis, a recurrent-event time-to-event model (e.g., Andersen–Gill) may be applied. Deaths occurring during the index hospitalization will be recorded as safety outcomes but will not be included in the primary time-to-event analysis, which focused on post-discharge events.

Changes in weight, NT-proBNP, and KCCQ scores over time will be evaluated using linear mixed-effects models with random participant intercepts, incorporating all available repeated measurements.

Safety endpoints (acute kidney injury, hypotension, electrolyte imbalance) will be summarized as counts (%) and compared using *χ*² or Fisher's exact tests.

The primary endpoint will be tested at a two-sided alpha level of 0.05. All secondary endpoints will be analyzed as exploratory; no formal multiplicity adjustment will be applied. For secondary and safety outcomes, effect estimates with 95% confidence intervals will be reported and interpreted cautiously.

#### Missing data handling

Variables with substantial missingness will not be excluded *a priori* if clinically important; instead, missingness patterns will be reported, and multiple imputation will be used under a missing-at-random assumption, with sensitivity analyses exploring departures from this assumption.

If warranted, subgroup analyses will be performed according to prespecified variables such as age, left ventricular ejection fraction, and other clinically relevant factors, and will be interpreted as exploratory.

### Monitoring

To guarantee data integrity and protocol adherence, an independent data-monitoring committee (IDMC) appointed by the sponsor will conduct rigorous oversight throughout the trial, performing periodic reviews of accrual, safety data, and key efficacy endpoints to ensure the study meets ethical, clinical, and data-management standards.

#### Data monitoring

Purpose of monitoring: to verify accuracy, completeness, and consistency of all study data and to detect errors or protocol deviations promptly. Monitoring will combine centralized remote review with on-site visits as needed.

#### Remote monitoring

Real-time review of study data via the electronic data-capture platform and online dashboards will allow early detection and correction of issues without disrupting trial conduct. Key elements include:

##### Data-entry review

Regular audits of accuracy and consistency to verify that all data have been correctly entered into the database.

##### Data-consistency checks

Automated logic rules within the data-management system flag missing, out-of-range, or inconsistent values.

##### Remote meetings

Scheduled virtual conferences between the study team and data-monitor staff to review data queries or potential protocol deviations and implement immediate corrective actions.

#### On-site monitoring

On-site monitoring will be conducted by dedicated monitors who will visit the center to ensure full adherence to the protocol. A comprehensive on-site review will be conducted at least once every 3 months; any deficiencies identified will be immediately communicated to site staff and corrective actions documented. Key activities include:

##### Document review

Verification of informed-consent forms, case-report forms, and laboratory reports to ensure accuracy and completeness of all study documentation.

##### Compliance verification

Confirmation that all study procedures conform to ethics-committee approvals, the clinical trial protocol, and applicable regulatory requirements.

##### Drug-use review

Verification of study-drug (including diuretics) accountability, administration, and documentation to ensure use adheres to the protocol.

##### Participant-follow-up audit

Review of visit logs to confirm that follow-up encounters occur on schedule and that data collection meets protocol specifications.

##### Safety surveillance

Evaluation of adverse event (AE) and serious adverse event (SAE) reporting and management to ensure participant safety.

##### Interim analyses and stopping guidance

No formal interim efficacy analysis is planned. The IDMC will periodically review recruitment, protocol adherence, and safety signals (e.g., acute kidney injury, hypotension, electrolyte abnormalities) and may recommend protocol modifications or trial suspension in case of unexpected safety concerns.

### Data access

Paper originals will be retained by the study sites in accordance with applicable regulations. Electronic files and data will be stored in a controlled network directory accessible only via authorized accounts using unique user IDs and passwords. This access system is not publicly available through direct links, ensuring that only trained and authorized research personnel who have completed data processing training can access the data. Access to the database will be strictly controlled through a secure user ID and password authentication system.

Data will be captured and reported via paper or electronic CRF; paper CRF must be signed by the investigator, who retains full responsibility for accurate source data and for permitting study-related monitoring, auditing, IRB review, and regulatory inspection. The sponsor (or designee) is responsible for data management, including quality checks. Unless local regulations or institutional policy mandate longer retention, investigators must retain all study records (including signed informed-consent forms) for 10 years post study completion; no documents may be destroyed or transferred to another location or third party without prior written approval from the sponsor.

## Ethics and dissemination

### Ethics approval

The study has been reviewed and approved by the Medical Ethics Committee of the First Affiliated Hospital of Xinjiang Medical University (Approval number: K202509-48) and registered at the Chinese Clinical Trial Registry (ChiCTR2500111518). Any subsequent amendments to the protocol, informed-consent form, or other study documents must be resubmitted to the same committee for review and formal approval or filing, as required by local policy. Annual progress reports will be submitted as mandated, and all applicable regulations of the committee and relevant authorities will be strictly followed throughout the trial.

Investigators/institutions consent to provide inspectors or monitors direct access to all pertinent records and to schedule discussions of any findings at a mutually convenient time; such audits may occur during or after study completion. Any protocol amendment must be submitted to the same committee (and to regulatory authorities) for review and approval before implementation.

### Protocol amendments

A protocol amendment is a written description of any change to, or formal clarification of, the protocol that may affect the conduct, potential benefit, or safety of trial participants. Amendments include modifications to the study objectives, design, population, sample size, procedures, or key administrative aspects. Changes to administrative details that do not materially influence study conduct or participant safety (e.g., telephone numbers, organizational assignments) are considered minor corrections or clarifications. All amendments require written approval from the sponsor and the Medical Ethics Committee of the First Affiliated Hospital of Xinjiang Medical University; regulatory authority approval will be obtained when mandated. If an amendment is required to protect participant safety, it will be implemented only after ethics-committee approval. Because formal review may require time, investigators may immediately institute any safety-driven measure that deviates from the protocol; such action must be reported to the ethics committee within 72 h.

### Informed consent

Prior to the collection of any personal information or trial data, a dated informed-consent form bearing the signatures of both the investigator and the participant must be obtained. Any modification to the consent document requires prior approval from the Medical Ethics Committee of the First Affiliated Hospital of Xinjiang Medical University. An ethics-approved copy of the form, together with the participant-signed original, must be retained in the designated trial binder; a duplicate signed copy must be provided to each participant. Investigators are responsible for ensuring that the entire consent process is conducted in accordance with Good Clinical Practice.

### Confidentiality

Each enrolled participant will be assigned a unique identifier; any medical record or dataset transmitted to the central study database will reference only this code, with all direct identifiers removed. Laboratory reports uploaded to the database must be de-identified prior to transmission, ensuring complete concealment of personal information. All biological specimens generated during the study will be disposed of after the planned analyses are completed, in accordance with institutional policy.

This study will collect personal information and data from patients; all precautionary measures will be taken to safeguard participant privacy. Identifiable information will be removed from datasets prior to entry into the central study database, and all participant- and site-level identifiers will be handled in a de-identified manner to ensure confidentiality.

All patients who meet eligibility criteria will be entered into a screening log and compared with those ultimately enrolled to assess selection bias. Only patients who provide written informed consent will be included; data will be collected per protocol during hospitalization and follow-up. Individuals who meet inclusion criteria but decline consent will be recorded solely with screening information.

All personally identifiable information (PII) will be stored on password-protected, access-controlled servers with audit trails, accessible only to authorized study staff. Upon study completion, PII will be securely deleted from the central database unless a longer retention period is required by law or institutional policy. During quality assurance and monitoring visits, monitors may access source medical records at the study site; participants will be informed of this practice in the informed consent form.

### Summary

This study addresses the clinical challenge of lacking a precise, quantitative guide for diuretic therapy in patients with acutely decompensated chronic heart failure. Current empirical diuresis often leads to either insufficient or excessive fluid removal; the former is closely associated with high rates of rehospitalization and mortality post-discharge, while the latter poses risks such as renal dysfunction and electrolyte disturbances (5, 7). Although precision volume management strategies guided by BIA have shown potential in assessing fluid status, high-level evidence from randomized controlled trials supporting its effectiveness in guiding diuretic therapy for heart failure is still lacking (11). This trial aims to systematically evaluate, through a rigorous design, whether the precision-based volume management strategy can improve short-term patient outcomes. The fundamental premise is to explore how an objective, non-invasive monitoring tool can be translated into a clinical protocol to optimize a key aspect of heart failure management.

This study is designed to make three significant contributions. First, this randomized controlled trial aims to generate high-quality evidence on whether a precision-guided volume management strategy reduces the 3-month composite endpoint of heart failure rehospitalization or all-cause mortality in patients hospitalized with acute decompensated chronic heart failure. Second, we aim to develop and validate an individualized, BIA-driven, in-hospital fluid management protocol, thereby providing an actionable strategy for achieving precision volume management. Finally, the findings will offer direct evidence for the development of prognosis-oriented, precision-based diuretic treatment pathways, enabling healthcare decision-makers, clinicians, and administrators to advance quality improvement in heart failure management.

The findings are expected to directly influence the treatment practices for hospitalized heart failure patients by demonstrating the clinical benefits of a precision volume management strategy to guide diuretic therapy, thereby promoting its standardized application in heart failure management. On a broader level, this study provides a paradigm for exploring precision medicine in heart failure, aiming to improve key hard endpoints through the integration of non-invasive monitoring technology with conventional pharmacotherapy. Ultimately, this research supports a shift towards more personalized, goal-directed models of heart failure care, offering new insights and tools to reduce the disease burden and improve long-term patient outcomes.

From a public health perspective, validating a protocol based on widely accessible technology like BIA could democratize precision medicine, allowing for standardized, high-quality volume management across different tiers of the healthcare system, thereby optimizing resource allocation.

### Strengths and limitations of the study

This trial represents the first randomized controlled design to validate the efficacy and safety of the precision volume management strategy in patients with acute decompensation of chronic heart failure. Its core innovation lies in establishing a structured, closed-loop “measure–assess–adjust–reassess” pathway, which integrates the quantitative threshold of fluid overload (overhydration, OH) measured by BIA into daily clinical decision-making. This protocol aims to enable precise, individualized, and standardized volume control, thereby optimizing diuretic therapy.

However, several limitations should be acknowledged. First, due to the nature of the intervention, which necessitates that treating physicians actively view BIA data to adjust diuretic dosages, blinding of the clinical team is not feasible. This open-label design may theoretically introduce performance bias. To mitigate this, the primary endpoint comprises objective “hard” outcomes (all-cause mortality and heart failure rehospitalization) that are less susceptible to subjective influence. Furthermore, all clinical events will be adjudicated by an independent CEC blinded to treatment allocation to ensure the integrity of the results.

Finally, the single-center design of this study may limit the external validity and generalizability of the findings to other populations or healthcare settings. Consequently, future multicenter randomized trials will be essential to confirm and extend our conclusions.
