# Supplementary material for: The GUIDE-HF protocol: a randomized controlled trial of bioelectrical impedance analysis-guided diuretic therapy on prognosis in patients with acutely decompensated chronic heart failure
Source: Front Cardiovasc Med. 2026 May 8;13:1798341. doi: 10.3389/fcvm.2026.1798341 (PMC13194452; doi:10.3389/fcvm.2026.1798341)
Supplement: Supplementary file 1 [file Datasheet1.docx]

**Supplementary File 1**

**BCM Operating Manual (Fresenius, Germany, CZ 3/12.07)**

1. Preparation:

1) Placement of the BCM device

Place the BCM on a stable, level surface, such as a non-metallic table or treatment cart.

2) Power Supply Assurance

The battery must be fully charged. If the battery level is insufficient, connect the device to an external power source via the power adapter.

3) Patient Preparation:

Prior to measurement, the patient must lie supine for 5-10 minutes to ensure fluid equilibrium;

Vigorous exercise and excessive food or fluid intake are prohibited within the two hours preceding the measurement;

Assess the patient for fever or diarrhea prior to measurement;

Remove conductive objects from the patient, such as watches and mobile phones;

During measurement, the patient must lie with limbs fully abducted and avoid contact with conductive surfaces, such as the metal side rails of the bed.

4) Preparation of Measurement Accessories

Prepare the electrode cables and disposable matching electrodes.

5) Preparation of the Measurement Environment

Maintain the ambient temperature at 22℃-26℃;

Exclude personal computers equipped with card readers and system test boxes from the environment.

1. Operating Procedures (as shown in the figure below):

1) Power on the device: Access the no-patient-data card interface. The startup screen will display the BCM logo and version number, along with the message "No chipcard."

2) Initiate a new measurement: Enter the sex, age, height, weight, systolic blood pressure, and diastolic blood pressure in sequence.

3) Verify patient information:

If the data are correct, press <confirm>; if the data are incorrect, press <back> to return to the previous level for correction.

1. Electrode Placement

The arms must not contact the body. The legs and feet should be separated by 12 inches. It is essential to ensure that the patient's arms and legs are fully separated.

*Hands*: The distal electrode (injector-red) is placed on the knuckles of the dorsal hand; the proximal electrode (receiver-black) is positioned at the imaginary midline traversing the wrist.

*Feet*: The distal electrode (injector-red) is placed on the toe joints of the dorsal foot; the proximal electrode (receiver-black) is positioned as precisely as possible at the imaginary midline traversing the ankle.

1. Initiation of Measurement

1) Upon confirming that the patient is correctly connected, press <start> to initiate the measurement.

2) The display of "Measuring……" indicates that the measurement is in progress; wait for the results.

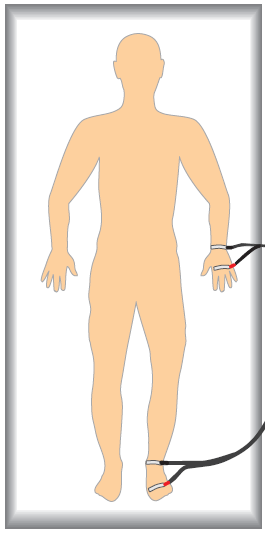


1. Display of Results:

The instrument emits two short beeps. When the waveform appears as shown in the figure, the measurement is complete. Press <continue> to view the results; the up/down keys are used to navigate the pages.

If an invalid waveform occurs, press <cancel> to repeat the measurement.

1. Precautions:

1) Prior to electrode application, wipe the skin contact area with alcohol to remove hand lotion, oil, or sweat, and allow the skin to dry completely before application. In cases of excessive body hair, the skin at the electrode contact site must be shaved.

2) The skin at the electrode contact site must be intact, without abrasions, erythema, or edema.

3) A minimum waiting period of two minutes is required after electrode placement before initiating measurement.

4) Disposable electrodes must be placed at the designated sites on the ipsilateral hand and foot, with an inter-electrode distance of at least 3 cm.

5) Electrodes are strictly for single-use only and must not be reused after removal.

6) The electrode cable must not be twisted, knotted, or coiled. During measurement, it must not contact the ground or any metallic objects or personnel.

7) During measurement, the patient must remain motionless and silent, maintaining a state of relaxation.

8) During measurement, the patient's limbs must be abducted and must not touch each other; the operator must not carry high-frequency electronic devices, such as mobile phones or computers.
